# Association between the *TNFRII *196R allele and diagnosis of rheumatoid arthritis

**DOI:** 10.1186/ar1777

**Published:** 2005-06-29

**Authors:** Vincent Goëb, Philippe Dieudé, Olivier Vittecoq, Othmane Mejjad, Jean-François Ménard, Marlène Thomas, Danièle Gilbert, Patrick Boumier, Sophie Pouplin, Alain Daragon, Patrice Fardellone, François Tron, François Cornélis, Xavier Le Loët

**Affiliations:** 1 Rheumatology Department, University Hospital of Rouen, Rouen, France; 2 Inserm U519, IFRMP 23, Faculty of Medicine, Rouen, France; 3 GenHotel, University of Evry-Paris VII, Faculty of Lariboisière-Saint Louis, Evry-Genopole and Unité de Génétique clinique, University Hospital of Lariboisière, APHP, Paris, France; 4 Biostatistics Department, University Hospital of Rouen, Rouen, France; 5 Rheumatology Department, University Hospital of Amiens, Amiens, France

## Abstract

Tumour necrosis factor (TNF)-α plays a key role in the pathogenesis of rheumatoid arthritis (RA). It binds to two receptors, namely TNF receptor (TNFR)I and TNFRII. Several studies have suggested an association between *TNFRII *196R/R genotype and RA. The objective of the present study was to evaluate the predictive value of the *TNFRII *196R allele for RA diagnosis and prognosis in a cohort of patients with very early arthritis. We followed up a total of 278 patients recruited from the community, who had swelling of at least two joints that had persisted for longer than 4 weeks but had been evolving for less than 6 months, and who had not received disease-modifying antirheumatic drugs or steroid therapy. At 2 years, patients were classified according to the American College of Rheumatology criteria. All patients were genotyped with respect to *TNFRII *196M/R polymorphism. Radiographs of hands and feet (read according to the modified Sharp method) and the Health Assessment Questionnaire were used to quantify structural and functional severity. The cohort of 278 patients was found to include 156 and 122 RA and non-RA patients, respectively. The *TNFRII *196R allele was found to be associated with RA (*P *= 0.002). However, progression of radiographic severity and Health Assessment Questionnaire scores over 1 year did not differ between carriers of the 196R allele and noncarriers. Our findings suggest that the *TNFRII *196R allele may be associated with RA diagnosis but that it does not predict early radiographic progression or functional severity in patients with very early, unclassified arthritis.

## Introduction

Rheumatoid arthritis (RA) is the most common chronic inflammatory joint disease, and it can lead to progressive joint destruction, deformity and severe disability. Early diagnosis of RA and timely initiation of disease-modifying antirheumatic drugs (DMARDs) are necessary to limit joint damage and optimise the functional outcome (i.e. the concept of a 'window of opportunity') [1,2]. No diagnosis criteria for RA are yet available, the 1987 American College of Rheumatology (ACR) criteria being classification criteria [3]. With the overall objective being to manage patients better, identification of markers that would allow one to establish a diagnosis of RA at the very beginning of the disease process remains an important goal. Certain autoantibodies have been reported to be specific for RA [4] and thus may help in the diagnosis of RA. Autoantibodies against cyclic citrullinated peptides (anti-CCP) are specific for RA but lack sensitivity; this contrasts with rheumatoid factor, which has strong sensitivity but low specificity for RA. Recently, a study conducted in blood donors [5] showed that positivity for IgM rheumatoid factor and anti-CCP may precede the clinical manifestations of RA. However, although concomitant positivity of both markers has been shown to be highly predictive of a diagnosis of RA, it has a low sensitivity (<50%) [6,7]. Thus, new RA diagnosis markers are needed, such as autoantibody populations and/or genetic markers. The latter have the particular advantages of being present from the onset of the disease and of remaining unchanged by therapy. To date, the only genetic susceptibility factor identified for RA is HLA-DRB1. This association is restricted to HLA-DRB1 alleles encoding a specific conserved amino acid sequence referred to as the shared epitope [8]. The predictive value of the shared epitope alleles for diagnosis of RA was studied in a cohort of 680 patients with early unclassified arthritis and was found to be lower than expected [9]. The contribution of HLA to the overall genetic risk has been estimated to range from 30% to 50% [10]. These data suggest that non-HLA genes are involved in RA susceptibility and could represent a very helpful tool for diagnosis of RA. Genome scans have implicated 1p36 as a susceptibility locus for RA [11,12], and *TNFRII*, which encodes the tumour necrosis factor (TNF)-α receptor (TNFR)II, is located within this locus [13]. Recent studies have reported an association between the *TNFRII *196R/R genotype and familial RA in UK and French Caucasian populations [14,15] and RA in the Japanese population [16]. In the UK and French populations, the association was restricted to familial RA [14,15]. However, a case-control study conducted in the Swedish population [17] failed to replicate the association between RA and the *TNFRII *196M/R polymorphism. That study revealed that RA patients carrying the *TNFRII *196R allele were significantly younger at disease onset than were those homozygous for the *TNFRII *196M allele.

Recent studies have reported conflicting results concerning the value of the *TNFRII *196R allele as a marker of RA severity [18-20]. Glossop and coworkers [19] reported no association between this single nucleotide polymorphism and functional or radiological RA severity. van der Helm-van Mil and coworkers [20] recently reported similar findings in a study based on a comparison of the extremes of phenotypes. Constantin and colleagues [18] found a worse Health Assessment Questionnaire score in RA patients carrying the *TNFRII *196R allele. Taking those data into account, the aim of this study was to assess the contribution of the *TNFRII *196R allele, alone or in combination with HLA-DR1/DR4 alleles, in predicting RA diagnosis and prognosis in a community-based cohort of patients with very early arthritis (VErA study [6]).

## Materials and methods

### Patients

The VErA cohort comprises 314 patients with early inflammatory arthritis who were prospectively recruited between October 1998 and January 2002 in two French regions: the entire province of Upper Normandy (1,800,000 people) and the metropolitan area of Amiens (300,000 people). All private rheumatologists and those running rheumatology clinics in the five hospitals of these areas were contacted regarding the project. In parallel, most general practitioners were asked to participate. All these physicians were encouraged to notify and refer all patients with inflammatory polyarthritis to one of the four hospital clinics organized to conduct assessments (Amiens, Evreux, Le Havre and Rouen). To contact as many patients as possible, and so obtain a representative sample of these regions, a large publicity campaign was conducted each year via the news, radio and TV media. Patients were required to have swelling of at least two joints that had persisted for longer than 4 weeks but had been evolving for less than 6 months, and who had not received DMARDs and/or steroid therapy before inclusion. Excluded were patients younger than 18 years, those with a history of inflammatory back pain, and pregnant or nursing women. The mean (± standard deviation) age of the 314 VErA patients was 51.7 ± 14.5 years (range 19–84 years) and the female/male ratio was 2.17. All were European Caucasians. No information was available concerning the past history of RA in first-degree and second-degree relatives.

Every 6 months, VErA patients were evaluated and classified using the ACR 1987 criteria for RA [3]. Only those VErA patients with well defined RA and unclassified inflammatory polyarthritis were followed up. Thus, VErA patients with well defined non-RA rheumatism were included in the study but were not followed up, and so radiographs from the follow-up period were not available for the majority of them. The same therapeutic approach was applied in all patients; specifically, hydroxychloroquine was tried first and, in the case of nonresponse, patients were switched to methotrexate. None received any biologics during the study.

At baseline and during the follow-up period we collected clinical (Disease Activity Score, the French version of the Health Assessment Questionnaire [F-HAQ]), biological (erythrocyte sedimentation rate, C-reactive protein, autoantibodies), genetic (HLA-DR typing, *TNFRII *196M/R polymorphism genotyping [see below]) and radiological data (see below). Before entry into the protocol, each patient gave his or her written consent after receiving verbal and written information regarding the nature, duration and purpose of the study. The protocol was approved by the Committee for Protection of Persons Participating in Biomedical Research of Rouen (French law 88–1138; 20 December 1988).

### Radiographic assessments

Radiographs of hands and feet were performed at inclusion and every 6 months during the follow-up period. Radiographs were scored chronologically by two independent rheumatologists (OM and PF) according to the van der Heijde/modified Sharp method [21]. The total radiographic damage score (range 0–448) was used to quantify progression of structural damage for the whole cohort and for RA patients.

### *TNFRII *196M/R polymorphism genotyping

Genomic DNA used for genotyping was extracted from EDTA anticoagulated peripheral blood leucocytes using standard methods. *TNFRII *196M/R polymorphism genotyping was performed using PCR-RFLP (polymerase chain reaction-restriction fragment length polymorphism) with the enzyme *Nla*III, as previously described [22]. The substitution at codon 196 (i.e. ATG [methionine] → AGG [arginine]) eliminated the *Nla*III restriction site. Each genotype was interpreted independently by two individuals (VG and PD) who were unaware of the underlying disease process. HLA-DRB1 shared epitope genotypes were not available.

### Statistical analysis

Taking into account the previously reported low frequency of the *TNFRII *196R/R genotype in the French Caucasian population and the suggested association between the *TNFRII *196R allele and severity of RA [18], Fischer's exact test and Student's t-test were performed to test for an association between the *TNFRII *196R allele and RA diagnosis and age at onset of RA. To determine the potential relationship between *TNFRII *196R allele and progression of structural damage and functional severity, Mann–Whitney test was performed comparing the variation in radiological score over 1 year of follow up and comparing the progression of F-HAQ score over the same period, both for the whole cohort and for RA patients. All of these statistical analyses were also performed for HLA-DR status, considered alone or in combination with the *TNFRII *196R allele. *P *< 0.05 was considered statistically significant.

### Hardy–Weinberg equilibrium check

The Hardy–Weinberg equilibrium of the *TNFRII *196M/R polymorphism was investigated using a χ^2 ^test with one degree of freedom.

## Results

### Main characteristics of the 314 patients included in the VErA cohort

Baseline characteristics of the VErA cohort, subdivided according to diagnosis as defined using ACR criteria, are summarized in Table 1. From this cohort, 278 patients were studied. According to ACR criteria, 156 patients were classified as having RA and 122 as having non-RA disease, including well defined (*n *= 55) and undifferentiated (*n *= 67) arthritides.

### HLA-DR status and diagnosis, functional severity and early progression of joint damage of RA

Among RA patients, 13% and 33% were heterozygous for at least the HLA-DR1 and HLA-DR4 alleles, respectively. HLA-DR1 and/or HLA-DR4 status (i.e. presence of at least one HLA-DR1/DR4 allele) was not found to be associated with RA diagnosis (*P *= 0.051; positive predictive value [PPV] 62.3%, negative predictive value [NPV] 49%; odds ratio [OR] 1.59, 95% confidence interval [CI] 0.99–2.57) and RA functional severity (*P *= 0.182). However, positivity for at least one HLA-DR1 and/or HLA-DR4 allele was found to be associated with early progression of joint damage both for the whole cohort (*P *= 0.011) and for the subgroup of patients with RA (*P *= 0.0012).

### *TNFRII *196M/R genotypes, 196R allele frequencies and diagnosis of very early RA

A total of 283 VErA patients were genotyped for the *TNFRII *196M/R polymorphism and, of these, five genotypes were uninterpretable. Indeed, DNA material for 31 patients was not available either because of patient refusal to participate in the genomic study or because of delayed inclusion of the last patients. The frequencies of the *TNFRII *196M/M, 196M/R and 196R/R genotypes were (respectively) 48.7%, 46.8% and 4.5% in RA patients, and 67.2%, 27.1% and 5.7% in non-RA patients. The frequencies of the *TNFRII *196R allele in RA and non-RA patients are shown in Table 2. The *TNFRII *196R allele was found to be associated with diagnosis of RA (*P *= 0.002; PPV 66.6%, NPV 51.9%; OR 2.158, 95% CI 1.284–3.641). Comparison of the frequency of the *TNFRII *196R allele in RA with that in the subgroup of patients with undifferentiated arthritides revealed that this allele was also significantly associated with RA diagnosis (*P *= 0.012). There was no statistically significant difference in age at onset of RA between *TNFRII *196R allele carriers and noncarriers (age 52.72 years versus 51.23 years, respectively; *P *= 0.40).

### *TNFRII *196R allele and early progression of joint damage

Radiographs of hands and feet were available for 237 patients from the VErA cohort. Table 3 shows the baseline and 1-year radiographic scores, and progression of the radiographic scores according to the absence or presence of the *TNFRII *196R allele, both for the whole cohort and for the subgroup of patients with RA. At baseline and after 1 year of follow up the radiographic damage scores did not differ statistically between *TNFRII *196R allele carriers and noncarriers. Progression of the radiographic score did not differ between 196R allele carriers and noncarriers (*P *= 0.98 [whole cohort] and *P *= 0.92 [RA patients]).

### *TNFRII *196R allele and functional severity of RA

Table 4 shows the baseline and 1 year F-HAQ scores as well as variation in F-HAQ index over the 1-year follow-up period, stratified by absence or presence of the *TNFRII *196R allele. At baseline and after 1 year of follow up, the F-HAQ scores did not differ statistically between *TNFRII *196R allele carriers and noncarriers. Progression of the F-HAQ did not differ between carriers of the 196R allele and noncarriers (*P *= 0.31 [whole cohort] and *P *= 0.70 [RA patients]).

### Concomitant presence of TNFRII 196R allele and at least one *HLA-DR1/DR4 *allele

Concomitant presence of *TNFRII *196R allele and at least one *HLA-DR1/DR4 *allele was found to be associated with RA diagnosis (*P *= 0.012; PPV 71%, NPV 47.4%; OR 2.2, 95% CI 1.16–4.32) but not with early progression of joint damage, both for the whole cohort (*P *= 0.806) and for RA patients (*P *= 0.802), or with functional severity, both for the whole cohort (*P *= 0.285) and for RA patients (*P *= 0.587).

### Hardy–Weinberg equilibrium checks

We found the *TNFRII *196M/R genotype distributions in the VErA patients to be in Hardy–Weinberg equilibrium.

## Discussion

The aim of this study, conducted in a French Caucasian cohort of patients with very early arthritis (VErA cohort), was to evaluate the possible association between presence of the *TNFRII *196R allele and RA diagnosis and prognosis. The results of this prospective longitudinal study suggest an association between the *TNFRII *196R allele and diagnosis of RA. Patients carrying the *TNFRII *196R allele were more likely to develop RA than were noncarriers (*P *= 0.002). The *TNFRII *196R allele appears to be significantly associated with RA but not with arthritis in general (*P *= 0.012) and might discriminate between RA and non-RA arthritis. However, the age at onset of RA was not statistically different between *TNFRII *196R allele carriers and noncarriers. The diagnostic value of the *TNFRII *196R allele was unremarkable in the present study because the PPV and NPV were only 66.6% and 51.9%, respectively. This result is not surprising. Indeed, in few genes outside the HLA region has the association with development of RA been convincing. Although the 'shared epitope' alleles of HLA-DRB1 have an OR of about 2–2.5, they appear to have little diagnostic value and are not routinely used in the diagnosis of RA because they are not part of the ACR criteria for the diagnosis of RA. Taking into account a single genetic marker for RA diagnosis and/or prognosis will lead to weak performance. Several studies have shown the importance of examining several markers concomitantly for predicting RA diagnosis. In this respect, concomitant positivity for rheumatoid factor and anti-CCP ensure a diagnosis of RA. Furthermore, combined positivity for anti-CCP and a genetic marker (HLA-DRB1) in 'healthy individuals' is strongly associated with future development of RA [23]. *TNFRII *196R allele could be part of a diagnosis/prognosis algorithm and could be combined with other factors to improve the PPV and NPV for RA diagnosis.

The frequencies of the *TNFRII *196R allele observed in the present study are not statistically different from the previously reported frequencies in the UK and the French RA populations (range 20–27% for non-familial RA, and 27–37% for familial RA) [14,15]. Moreover, the familial status of the RA patients in the VErA cohort is unknown.

Our study's contribution is a comparison of the *TNFRII *196R allele frequencies between community recruited RA and non-RA patients with very early arthritis and similar clinical manifestations at inclusion. However, because of the type of recruitment of the VErA cohort, RA patients who usually require corticosteroids at the onset of the disease were not included in the present study, which led to exclusion of the more severe forms of the disease. Thus, the functional severity and RA structural damage observed may be of lesser magnitude than in studies conducted in populations recruited from hospitals.

Although the present findings revealed statistical significance between the *TNFRII *196R allele and RA diagnosis, independent studies are needed before it may be concluded that there is an association between the *TNFRII *196R allele and RA diagnosis. Indeed, in a complex disease such as RA, a particular combination of genetic and environmental factors is needed for the disease to develop. Hence, the probability of developing the disease is greater when those risk alleles are present. However, the genetic contribution of each allele to the risk for development of the disease is unknown and probably modest. The problem is further complicated by the fact that many of these alleles interact with other genes in the background as well as with environmental factors. Thus, the use of one genetic marker to predict diagnosis and/or prognosis in a complex disease is probably limited by the low contribution of that marker. Nevertheless, a particular combination of various genetic markers could confer significant risk that may represent a powerful tool in predicting diagnosis and/or prognosis. In the case of the *TNFRII *196R allele, the relative risk observed was under 3, suggesting the involvement of other genetic markers. However, even though concomitant presence of *TNFRII *196R allele and at least one HLA-DR1/DR4 allele was also found to be associated with RA diagnosis (*P *= 0.012), their combination did not improve upon the diagnostic accuracy of the *TNFRII *196R allele alone.

We also tested the hypothesis that there is an association between the *TNFRII *196R allele and RA structural severity in our cohort of patients with very early arthritis. The results show that the progression of the radiographic damage after 1 year of follow up did not differ between the whole group of patients and the subgroup of patients with RA, whether the *TNFRII *196R allele was carried or not. Previous studies also reported no relationship between the 196R allele and progression of joint damage [18-20]. In contrast, positivity of at least one HLA-DR1 and/or HLA-DR4 allele was found to be associated with early progression of joint damage in RA patients (*P *= 0.0012), as previously described [24], whereas their concomitant presence with *TNFRII *196R allele was not (*P *= 0.802).

As was previously reported by van der Helm-van Mil and coworkers [20], we observed a lack of association between the *TNFRII *196R allele and the functional severity of RA, which is in disagreement with the findings reported by Constantin and coworkers [18]. There may be several explanations for these discrepancies, including the heterogeneity of the studied population, differences in the selected outcome criterion between studies, and the influence of treatment, notably with DMARDs and biologics, on outcome. In this respect, one should recall that early and aggressive treatments were reported to affect the relationship of HLA class II alleles with progression of joint damage in RA [25]. Thus, we cannot exclude the possibility that DMARDs and biologics interfere with the possible association between presence of the *TNFRII *196R allele and RA structural or functional severity. Because the VErA patients were all treated with the same DMARD schedule, our data support the hypothesis that the *TNFRII *196R allele is not associated with functional severity of RA. Nevertheless, after only 1 year of follow up it is probably premature to conclude that there is no association between *TNFRII *196R allele carriers and noncarriers and RA functional severity. Moreover, we investigated the ability of the *TNFRII *196R allele to predict rapid radiographic progression in patients with very early RA. Our study appears to show that *TNFRII *196R allele is not able to predict rapid radiographic progression in very early RA. However, because the kinetics of radiographic progression are heterogeneous among patients developing RA, we cannot exclude the possibility that the *TNFRII *196R allele can predict radiographic damage over a follow-up period of 3 or 5 years.

New insights were recently provided by recent data on the role of the TNFRII. Indeed, Morita and coworkers [22], using *TNFRII*-transfected HeLa cells activated with TNF-α, demonstrated that 196R-transfected cells transduce signals for IL-6 production more effectively than do 196M-transfected cells. It is now well established that IL-6 plays pathological roles in RA, and that blockade of IL-6 may be therapeutically effective in RA [26]. Recently, Till and coworkers [27], using transfected HeLa cell populations and immortalized fibroblasts from *tnfr1*^-/-^/*tnfr2*^-/- ^double knockout mice, reported an altered induction of apoptosis and nuclear factor-κB pathway in the *TNFRII *196R allele transfected cells, which could also serve as an explanation for the association of this allele with increased susceptibility to RA. Moreover, patients with the *TNFRII *196R/R genotype were shown to have worse RA course and to be less responsive to TNF antagonist therapy [28].

## Conclusion

Our findings suggest that the *TNFRII *196R allele may be associated with RA diagnosis but that it does not predict early progression of structural damage and functional severity in patients with very early arthritis. Independent studies are required before it may be concluded that there is a definite association between the *TNFRII *196R allele and RA diagnosis.

## Abbreviations

ACR = American College of Rheumatology; CCP = cyclic citrullinated peptides; CI = confidence interval; DMARD = disease-modifying antirheumatic drug; F-HAQ = French version of the Health Assessment Questionnaire; IL = interleukin; NPV = negative predictive value; OR = odds ratio; PPV = positive predictive value; RA = rheumatoid arthritis; TNF = tumour necrosis factor; TNFR = tumour necrosis factor receptor.

## Competing interests

The author(s) declare that they have no competing interests.

## Authors' contributions

VG and PD carried out the molecular genetic studies with the help of MT, DG, FT and FC, and acquired, analyzed and interpreted the data. OV, OM, PB, SP, AD, PF, FT, FC and XLL made substantial contributions to the acquisition of clinical and radiological data and to the recruitment and the follow up of patients. OV and XLL also revised the article critically for important intellectual content. JFM participated in the design of the study and performed the statistical analysis. All authors read and approved the final manuscript.

## Figures and Tables

**Table 1:** Baseline characteristics of the 314 patients included in the VErA cohort (American College of Rheumatology criteria at 2-year follow up)

Parameter	RA (*n *= 176)	Non-RA (*n *= 138)
Age (years; mean ± SD [range])	52.3 ± 14.8 (20–84)	51 ± 14.2 (19–84)
Disease duration (months; mean ± SD [range])	4 ± 1.7 (0.9–6)	4.2 ± 1.7 (1–6)
Disease Activity Score (mean ± SD [range])	3.5 ± 1.3 (0.5–7.5)	2.5 ± 1 (0.4–5.9)
ESR (mm 1st hour; mean ± SD [range])	29.7 ± 25.4 (2–110)	21.8 ± 22.9 (2–110)
CRP (mg/l; *n *< 5, mean ± SD [range])	24.8 ± 34.8 (2–206)	15.8 ± 24.8 (1.5–128)
RF (IgM isotype; % of positivity)	35.2	7.9
Anti-CCP (% of positivity)	39.2	3.6
HLA-DR1 (%)	13	18.5
HLA-DR4 (%)	33	20.7

CRP, C-reactive protein; ESR, erythrocyte sedimentation rate; RA, rheumatoid arthritis; RF, rheumatoid factor; SD, standard deviation.

**Table 2:** *TNFRII *196R allele frequency, alone and associated with *HLA-DR *status, subdivided according to American College of Rheumatology criteria for diagnosis of rheumatoid arthritis

	*TNFRII *196R allele		*P*	Concomitant presence of *TNFRII *196R allele and at least one HLA-DR1/DR4 allele	P
				
Diagnosis	Absent	Present			
RA (*n *= 156)	76 (48.7 %)	80 (51.3 %)	0.002	44 (71 %)	0.012
Non-RA (*n *= 122)	82 (67.2 %)	40 (32.8 %)		18 (29 %)	

Using Fischer's exact test, the *TNFRII *196R allele (*P *= 0.002; positive predictive value [PPV] 66.6%, negative predictive value [NPV] 51.9%; odds ratio [OR] 2.158, 95% confidence interval [CI] 1.284–3.641) and its concomitant presence with at least one HLA-DR1/DR4 allele (*P *= 0.012; PPV 71%, NPV 47.4%; OR 2.2, 95% CI 1.16–4.32) were found to be associated with diagnosis of rheumatoid arthritis (RA).

**Table 3:** *TNFRII *196R allele and progression of the structural damage over the 1-yr follow-up

	Whole cohort			RA patients		
	
	*TNFRII *196R allele		*P*	*TNFRII *196R allele		*P*
				
	Absent (*n *= 132)	Present (*n *= 106)		Absent (*n *= 76)	Present (*n *= 80)	
Baseline radiographic score	2.00 (0–24)	2.50 (0–38)	0.34	2.00 (0–21)	3.00 (0–38)	0.42
1-year radiographic score	2.00 (0–36)	3.00 (0–38)	0.24	2.00 (0–36)	3.00 (0–38)	0.27
Radiographic score progression	0.00 (-1 to +15)	0.00 (0–17)	0.98	0.00 (-1 to +15)	0.00 (0–17)	0.92

The total radiographic score, calculated in accordance with the van der Heijde modified Sharp method, was used to quantify the progression of the structural damage for the whole cohort and for rheumatoid arthritis (RA) patients, whether or not they carried the 196R allele. Values are expressed as median (interquartile range).

**Table 4:** *TNFRII *196R allele and functional severity progression over the 1-year follow up

	Whole cohort			RA patients		
	
	*TNFRII *196R allele		*P*	*TNFRII *196R allele		*P*
				
	Absent (*n *= 132)	Present (*n *= 106)		Absent (*n *= 76)	Present (*n *= 80)	
Baseline F-HAQ score	0.63 (0.0–2.75)	0.88 (0.0–2.50)	0.07	0.88 (0.0–2.75)	1.13 (0.0–2.50)	0.50
1-year F-HAQ score	0.46 (0.0–2.50)	0.38 (0.0–2.38)	0.78	0.63 (0.0–2.50)	0.63 (0.0–2.38)	0.99
F-HAQ score progression	-0.13 (-2.0 to +1.0)	-0.25 (-2.38 to +0.88)	0.31	-0.13 (-1.88 to +1.00)	-0.38 (-2.38 to +0.88)	0.70

The French version of the Health Assessment Questionnaire (F-HAQ) score was used to quantify functional severity for the whole cohort and for rheumatoid arthritis (RA) patients, whether or not they carried the 196R allele. Values are expressed as median (minimum-maximum).
